# Seroprevalence of Natural and Acquired Immunity against the SARS-CoV-2 Virus in a Population Cohort from Two Chilean Cities, 2020–2022

**DOI:** 10.3390/v15010201

**Published:** 2023-01-10

**Authors:** Loreto Núñez-Franz, Muriel Ramírez-Santana, Paola Rubilar, Cecilia Vial, Mauricio Apablaza, Claudia González, Macarena Said, Kathya Olivares, Lina Jimena Cortés, Juan Hormazábal, Luis Canales, Pablo Vial, Gloria Icaza, Rubén Quezada-Gaete, Ximena Aguilera

**Affiliations:** 1 Departamento de Salud Pública, Facultad de Ciencias de la Salud, Universidad de Talca, Talca 3460000, Chile; 2 Public Health Department, Faculty of Medicine, Universidad Católica del Norte, Coquimbo 1780000, Chile; 3 Centro de Epidemiología y Políticas de Salud, Facultad de Medicina Clínica Alemana Universidad del Desarrollo, Santiago 7550000, Chile; 4 Instituto de Ciencias e Innovación en Medicina, Facultad de Medicina Clínica Alemana Universidad del Desarrollo, Santiago 8320000, Chile; 5 Facultad de Gobierno, Universidad del Desarrollo, Santiago 7550000, Chile; 6 Facultad de Economía y Negocios, Universidad de Talca, Talca 3460000, Chile

**Keywords:** seroprevalence, seroepidemiology, COVID-19, vaccines, immunity, antibodies, Chile

## Abstract

Background: Chile has achieved the highest coverage for vaccines against the SARS-CoV-2 virus worldwide. Objective: To assess the progression of immunity (natural and acquired by vaccine) in a cohort from two Chilean cities. Methods: Individuals (*n* = 386) who participated in three phases of population-based serial prevalence studies were included (2020–2021 and 2022). Presence of SARS-CoV-2 antibodies was measured in serum. Data including time of vaccination and type of vaccine received were analysed with descriptive statistics. Results: Seroprevalence was 3.6% in the first round and increased to 96.9% in the second and 98.7% in the third. In the third round, 75% of individuals who had received the basal full scheme were seropositive at 180 days or more since their last dose; 98% of individuals who received one booster dose were seropositive at 180 days or more, and 100% participants who received two boosters were seropositive, regardless of time since their last dose. Participants receiving mRNA vaccines had higher seroprevalence rates over time. Conclusions: The high vaccination coverage in Chile enabled the population to maintain high levels of antibodies. Vaccination boosters are essential to maintain immunity over time, which also depends on the type of vaccine administered.

## 1. Introduction

Since the end of the year 2019, the world has witnessed an unprecedented pandemic caused by the SARS-CoV-2 virus; more than six million deaths and over six hundred million cases were reported during the first 30 months of the COVID-19 pandemic [1]. Disease surveillance is crucial at all stages of an epidemic to guide control measures; clinical, epidemiological and pathogen surveillance are initially of interest, and surveillance of immunity takes on greater significance once an epidemic is more advanced. Serial seroprevalence studies enable the surveillance of the development of immunity (natural and/or acquired) through the quantification of IgG antibodies in the population [2,3,4]. The findings of the first population-based seroprevalence study in Chile showed heterogeneity in transmission within and between the urban centres studied, with social disparity determining the geographic distribution of the disease. The seroprevalence of COVID-19 antibodies was associated with factors such as education, overcrowding and population density [5].

Vaccines are the most efficient and effective preventive measure to control an epidemic. Vaccines for the SARS-CoV-2 virus have been developed very quickly with both commonly used and modern platforms, including vaccines based on inactivated viruses, viral proteins, viral vectors or viral genetic material (messenger RNA) [6]. Each type of vaccine has a distinct formulation, with different numbers of doses and spacing between doses specific to each vaccine. Moreover, the reported effectiveness of SARS-CoV-2 vaccines is also variable and ranges between 50% and 95% depending on the type of outcome measured, the type of vaccine and individual factors among the inoculated population [7,8,9,10].

The vaccination program in Chile began at the end of 2020 with healthcare personnel and advanced rapidly to wide coverage of the entire population via various heterologous vaccination schedules. Initially, Sinovac’s CoronaVac vaccine, which is based on an inactivated virus, was widely used. Pfizer-BioNTech’s BNT162b2, Oxford-AstraZeneca’s AZD1222 and CanSino Biologics’ Ad5-nCoV vaccines were added during the first year, and, finally, Moderna’s COVID-19 (mRNA-1273) was included by February 2022. A detailed description of the vaccines used in Chile in October 2021 (the time of the second evaluation in this study) is described elsewhere [11,12]. The general Chilean population was offered a second booster with a mRNA vaccine at the time of the third evaluation (April–May 2022).

During the first year of the SARS-CoV-2 pandemic, various seroprevalence studies were carried out. Several of them explored the natural infection in general population and healthcare professionals before the launch of the first round of COVID-19 vaccines [13,14,15]. These seroprevalence studies observed a decrease in natural immunity over time [16,17]. Moreover, the duration of immunity acquired by vaccines remains uncertain and depends on the type of vaccine and inoculation scheme [11,12,18]. It is not always possible to measure both types of immunity (natural and acquired) separately via simple determination of antibodies, as IgG antibodies to the nucleocapsid (IgG anti-N) and the spike proteins (IgG anti-S) must be measured separately [19]. However, the sequential measurement of the levels of antibodies in the same population sample, along with knowledge of the history of disease and vaccination, makes it easier to identify natural and acquired immunity and also helps to facilitate the identification of the factors associated with immunity. Indeed, hybrid seroprevalence follow-up studies have been carried out in various countries [10,20,21,22,23,24,25,26,27].

The purpose of this study was to establish the progression of humoral immunity against the SARS-CoV-2 virus and to evaluate the effective coverage of the vaccination plan among residents of the urban areas of two Chilean cities after two years of the pandemic and the implementation of a wide vaccination campaign. Additionally, we aimed to investigate the associations of immunity with vaccination status and clinical and socio-environmental factors.

## 2. Materials and Methods

The present study is part of three serial SARS-CoV-2 prevalence studies carried out in two cities in Chile (La Serena-Coquimbo and Talca) during the years 2020 (September–November), 2021 (October–November) and 2022 (April–May). The participants of this study were all individuals who participated in all three evaluations.

### 2.1. Sampling Procedures

**Figure 1:**
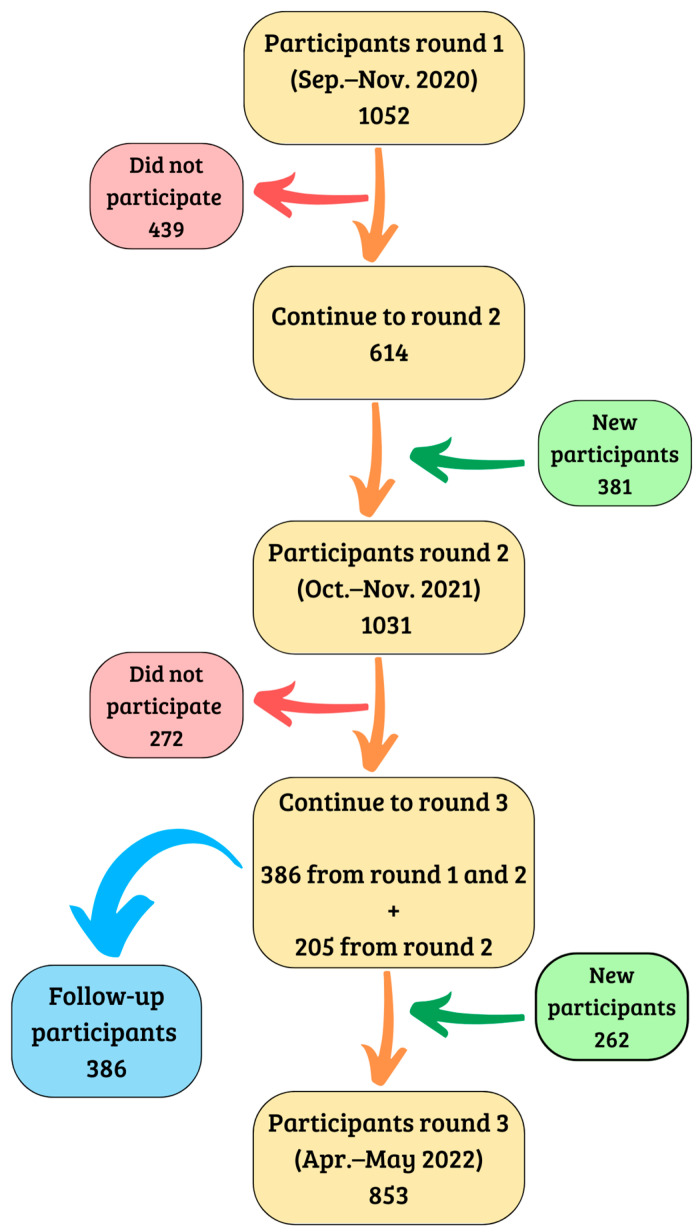
Scheme of the incorporation, loss and follow-up of the participants in the three rounds of the 2020–2022 seroprevalence study.

All population samples were randomly selected and were representative of both cities. The details of the sampling procedures for the first and second phases are described elsewhere [5,11,12]. Briefly, two-stage random sampling was carried out, stratified by census districts based on (1) block (random selection) and (2) home (systematic jump); all members 7 years of age or older within the sampled homes were invited to participate. The minimum numbers of participants required at all stages were calculated based on population sizes of 200,000 people (population of Talca) and 500,000 people (population of La Serena-Coquimbo), with an expected variance of 50%, considering a 95% confidence interval and 5% error. The minimum sample size required was calculated to be 384 individuals; this minimum was exceeded in all three stages of the present study for both cities (Figure 1).

### 2.2. Variables and Sources of Information

The sociodemographic variables measured were sex, age, educational level, nationality, health insurance, work activity, type of housing and overcrowding. The clinical and epidemiological variables registered were comorbidity history, risk factors, diagnosis and symptoms of COVID-19 and history of vaccination (vaccine received, date, dose, brand of vaccine and reasons for not receiving vaccine). Finally, we recorded the presence/absence and titres of IgG antibodies against SARS-CoV-2 in blood in the current and previous evaluations. The information was obtained by trained surveyors using a structured questionnaire and was recorded on the RedCap platform.

### 2.3. Field Work

Participants from previous studies were contacted, and an appointment was made to visit their homes. Surveys were conducted by trained medical students and nurses collected venous blood samples, which were stored in a cold chain. The samples were centrifuged, aliquoted and stored within 24 h at local laboratories. The sera were shipped, maintaining the cold chain, for analysis in a laboratory in Santiago, the capital city.

### 2.4. Laboratory Methods

The presence of anti-SARS-CoV-2 antibodies was determined in the serum of 386 individuals using different tests depending on availability. Three different strategies were used in the study due to the scarcity and variability in the available reagents, especially during the pandemic outbreak. The first serosurvey (2020) was performed using the Elecsys immunoassay (Roche^®^ with a cobas^®^ analyser, Basel, Switzerland) [5]. The Wantai SARS-CoV-2 Ab ELISA was used for the second serosurvey (2021) [11,12]. The third serosurvey (2022) was performed using an in-house validated ELISA [28]. All test results are expressed as dichotomous variables (positive/negative) to establish the proportion of enrolled seropositive individuals. A lateral flow immunoassay (Livzon^®^, Zhuhai, China; Cellex, Rockville, MD, USA) was used for adults in whom venepuncture failed or was contraindicated and for young children who refused venepuncture.

### 2.5. Statistical Methods

Seroprevalence was computed as the proportion of seropositive individuals expressed as a percentage of the total number of participants. Seroprevalence was also adjusted according to the sensitivity and specificity of the tests used, following the recommendations of Rogan and Gladen [29] using the Epitools calculator [30]. The data were analysed with descriptive statistics and corrected based on the sensitivity and specificity of the tests, including the variable time of vaccination, according to the types of vaccine received. The time elapsed since the last vaccination was calculated as the number of days from administration of the last vaccine to the date of sample collection.

Delta (days) = (sample collection date − date of the last administered vaccine). Kaplan–Meyer survival analysis was performed to show the loss of antibodies over time.

Data were analysed using STATA statistical software (StataCorp. 2017, Stata Statistical Software: Release 15; StataCorp LLC, College Station, TX, USA).

### 2.6. Ethical Considerations

The study protocol for each phase was approved by the Ethics Committees of the Universidad de Talca and the Facultad de Medicina of the Universidad Católica del Norte. Informed consent was obtained from all subjects; if subjects were under 18, written parent or legal guardian consent was obtained, and the children also signed an assent form.

## 3. Results

### 3.1. Characteristics of the Participants

A total of 386 subjects from two cities participated in all three rounds of this serial seroprevalence study (52.3% from Talca) (Figure 1). Most participants (67.4%) were female, 40 years of age or older (68.9%) and Chilean (99.5%); 22.2% of participants had professional education, and 85.8% had public health insurance (Table 1).

In total, 22.8% (88/386) of participants reported having ever been diagnosed with COVID-19 by PCR, and 2.3% of these individuals (2/88) reported two COVID infections.

### 3.2. Vaccine

At the time of the last evaluation, 98.4% individuals had at least one vaccine. Specifically, 0.3% had only one dose; 7% had two doses or full baseline; 55% had one booster, and 36% two boosters. The most frequent schemes for the entire sample were Pfizer–Pfizer–Pfizer (24.5%), followed by Sinovac–Sinovac–Pfizer (21.6%) and Sinovac–Sinovac–Astrazeneca–Pfizer (15%; Table 1).

In the second round (October–November 2021), 94.4% of all subjects had received either the basal scheme (47.2%) or the basal scheme plus one booster (47.2%). In the third round (April–May 2022), 61.9% of participants had received either the basal scheme (6.7%) or the basal scheme plus one (55.2%) or two boosters (36.0%; Table 2).

### 3.3. Seroprevalence

In the first round (September–November 2020) of this study, the seroprevalence rate was 3.6%. In the second round (October–November 2021), the seroprevalence rate was 96.9%. At least 96.7% of the individuals in the 20-to-over-70 age groups had antibodies against SARS-CoV-2. The seropositivity rate of the 10–19 age group was 91.7%, and children less than 10 years old had a seroprevalence rate of 50%. In the third round (April–May 2022), the seroprevalence was 98.7%, and 100% of 40 to 70-year-olds were seropositive (Table 2). The adjusted seroprevalence rates based on the sensitivity and specificity of the tests were not significantly different from the raw seroprevalence data; adjustment could not be performed for the second round due to the low number of negative cases (*n* = 12).

**Figure 2:**
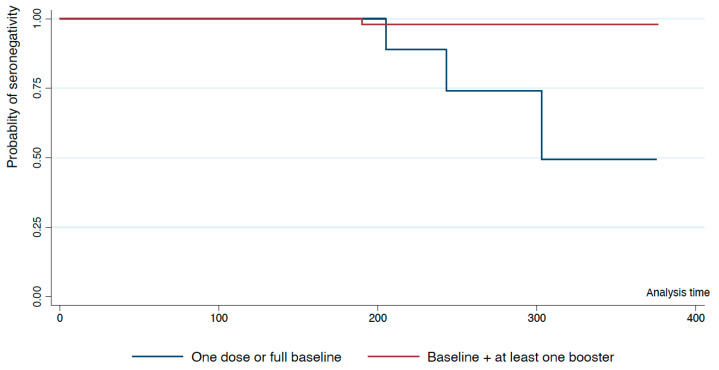
Kaplan–Meyer analysis shows probability of antibody loss over time, measured in days, depending on the dose of vaccine received.

Next, we analysed the seroprevalence according to the time elapsed since the last dose of vaccine. First, using Kaplan–Meier survival analysis, we explored the evolution of seronegative participants across time based on the number of doses administrated. The probability of losing seropositivity is 50% after 300 days since last vaccine for individuals with one dose or full baseline. On the other hand, individuals with at least one booster experience a marginal loss of seropositive starting 200 days after the last vaccine administrated (Figure 2).

**Figure 3:**
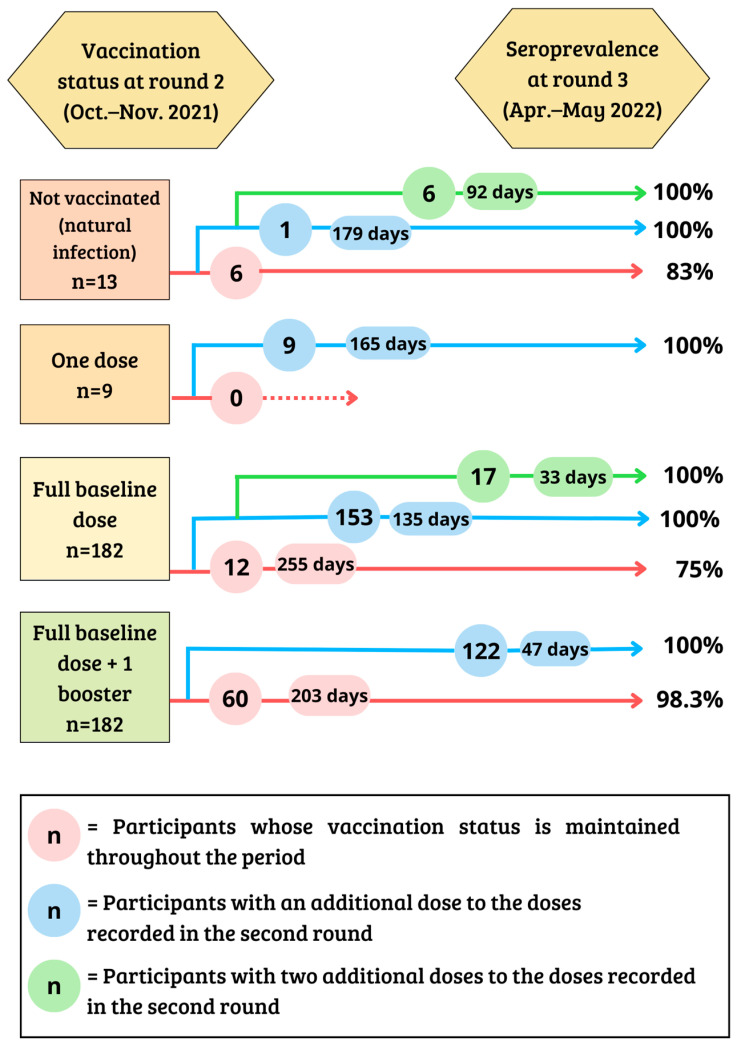
Seroprevalence rates in participants who did not receive another vaccine during the period and participants who received one (or two) additional doses during the period between rounds 2 and 3, respectively, according to second-round vaccination status and mean number of days since the last dose.

In the second round, 98.9% of participants with the complete basal scheme had antibodies against SARS-CoV-2 at 180 or more days since their last dose of vaccine. However, in the third round (April–May 2022), the seropositivity among participants who did not receive any booster after the full baseline scheme dropped to 75%. In the third round, 98.4% of participants who received the complete basal regimen plus one booster was seropositive after 180 days or more. In contrast, 100% of the participants who received two boosters (at least 15 to 179 days ago, respectively) were seropositive. All participants who received the basal scheme plus two boosters had antibodies against SARS-CoV-2, regardless of the time since their last dose (Table 3 and Figure 3).

Figure 3 presents the seropositivity rates of the participants in the third round of the study according to the doses of vaccine received between the second and the third rounds (Figure 3). The unvaccinated participants had a seropositivity rate of 83%. However, 100% of the participants who received one or two doses were seropositive, regardless of the average number of days since their last vaccine was administered. A similar scenario was observed in participants who had received the full basal scheme or the full basal scheme plus one booster. In these groups, the addition of one or two boosters increased the rate of seropositivity from 75% to 100% and 98.3% to 100%, respectively. In summary, regardless of vaccination status in the second round, the administration of one or two more doses between the second and third rounds resulted in 100% seropositivity.

Overall, 92.2% of participants were seronegative, seropositive, and seropositive for SARS-CoV-2 antibodies in rounds 1, 2, and 3, respectively (Table 4). In general, high seropositivity was observed in the second and third rounds for schemes that had one or two boosters. Nonetheless, two participants who received a basal scheme of CoronaVac were seronegative in the second round; these individuals had an average of 75 days since their last dose of vaccine but converted to seropositive after receiving the AstraZeneca booster. The lowest rate of seropositivity in the three rounds was observed for the group who received the full basal scheme with CoronaVac (63.2%) in the third round; 88.5% of this group were seronegative, exceeding 210 days since their last dose. A similar scenario was observed for one participant who received the complete baseline regimen with AstraZeneca; this patient was seronegative in the third round with a time elapsed since their last vaccine of more than 300 days.

One participant was seronegative in the third round, even though he/she received a complete basal scheme and a Pfizer booster. This participant did not receive boosters between the second and third rounds of this study, with 190 days from their last vaccination in the third round; this individual reported chronic kidney disease and obesity and had no diagnosis of COVID-19 during follow-up.

## 4. Discussion

The purpose of this study was to establish the progression of humoral immunity against the SARS-CoV-2 virus and to evaluate the effective coverage of the vaccination program among the residents of the urban areas of two Chilean cities after two years of the pandemic and the implementation of a wide vaccination campaign. We observed a high seroprevalence of antibodies against SARS-CoV-2 among the participants in the last round of this three-round study, which was mainly due to the wide coverage of vaccination campaigns based on heterologous technology in Chile. Administration of one or two booster doses of any vaccine between the second and third rounds of the study always resulted in 100% seropositivity, regardless of vaccine status in the second round. These results are consistent with the high vaccination rate in the country [31].

No other seroprevalence studies of a population-based cohort have reported the presence of SARS-CoV-2 antibodies annually; this study assessed the rates of seropositivity due to natural infection in the initial year of the pandemic and subsequently in the first and second years of the vaccination campaign. During the first phase of this study (September–November 2020), our research group verified that the risk of infection was related to social determinants of health [5]; these findings were confirmed by Mena et al. [32] and confirmed by de Oliveira et al., in Mato Grosso, Brazil [33]. However, this social disparity was mitigated by the deployment of the vaccination campaigns, which achieved high coverage in a short period of time, as demonstrated by the high prevalence of serum antibodies in both the second and third rounds of this study. This advanced vaccination coverage occurred thanks to the organization of primary healthcare in Chile. The national immunization program in Chile has universal coverage and reaches the population through family health centres and rural health posts under municipal administration. For the vaccination campaign against SARS-CoV-2, a mass communication campaign was carried out, and vaccination centres were set up in schools, sports centres, squares, or other places that could receive the population on a massive scale. Additional health personnel were temporarily hired for the vaccination program and cold-chain resources were reinforced. Similarly, Bastos et al. reported that vaccination programs in primary care allowed the most vulnerable municipalities to be protected in Brazil [34].

In our study, the proportion of the population vaccinated with the complete basal scheme in November 2021 was 83%, that is higher than that reported in other investigations. Moreover, the proportions of the population with SARS-CoV-2 antibodies in the second and third rounds of our research were higher than reported anywhere else (96.8% and 98.7%, respectively). In a convenience sample in the USA in February 2022, 81.3% of participants had antibodies due to infection [35] and in a study of blood donors reported a combined seroprevalence (induced by infections or vaccine) of 94.7% (95% CI, 94.5%–94.9%) in December 2021 [10]. In the UK, Hall et al. that found 27% of health workers had antibodies due to natural infection and that the seroprevalence rate due to vaccination for this group increased to 95% by the end of 2021 [26]. In Navarra (Spain), Castilla et al. reported a 92.7% seroprevalence rate of IgG anti-S (most probably due to vaccination) and 58.9% seroprevalence of IgG anti-N (most probably due to infection) in May 2022 [36].

Unvaccinated participants had a seroprevalence of 46.2% in the second round and 85.7% in the last round, which reflects the progression of the pandemic. One study reported a seroprevalence of 32% in unvaccinated people in Germany in July 2021 [25], while a rate of 58.9% was observed in Navarra, Spain, in May 2022 [36]. Although the number of unvaccinated participants in our study was quite low (*n =* 13 in the second round and *n =* 6 in the third round), this may reflect the low proportion of people who were infected over the first two years of the pandemic. If we consider the number of officially reported new cases of COVID-19, the cumulative incidence of infections was 10.8% at the time of the second round and 19.7% at the time of the third round (reported rates of 10,782 and 19,751 per 100,000 inhabitants, respectively) [37,38]. The first round of this seroprevalence study indicates that the actual prevalence of infections was 3 to 4 times higher, probably given the proportion of asymptomatic cases and people who did not seek healthcare [5]. Thus, the seroprevalence rate is 4.2–4.3 times higher than the reported cumulative incidence of infection. Similar figures were reported by a study carried out in Germany [25], which estimated that the relationship between seroprevalence and cases reported by surveillance varied over time and among districts, by a factor of between 2.2 and 5.1 at the end of 2020 and, subsequently, by between 1.3 and 2.9 in June 2021.

Despite assessing a small cohort, one strength of this study is that we detected the prevalence of antibodies in participants who received different vaccination schemes and counted the days since their last dose. Thus, we conclude that the lowest seroprevalence was achieved among participants who were vaccinated with the two doses of the complete basal scheme (without a booster) and whose average period since the last dose was over 8 months. This result agrees with Sendi et al. [39], who observed a decrease in antibody titres 150–200 days since the second dose of vaccine in Switzerland and emphasizes the need for booster doses to strengthen immunity. In the UK, Hall et al. found that antibody titres varied depending on the type of vaccine received and the time elapsed since the last dose [26]. The same authors also showed that the immunity acquired after infection was increased by at least one dose of vaccine, although the follow-up only lasted for up to 18 months. This finding becomes more important in the presence of new variants that could escape the immunity provided by infection or vaccines produced with the original strain of the SARS-CoV-2 virus [40,41]. Additionally, among the participants in our study who received the baseline regimen, 16% of the seropositive individuals in the second round who received Sinovac’s CoronaVac and the single dose of AstraZeneca were seronegative in the third round, whereas all individuals who received a Pfizer booster vaccine remained seropositive. These results are in line with the report by Sauré et al. [18], who observed a decrease in seropositivity over time in people from Chile who received the CoronaVac vaccine but not in those who received the Pfizer vaccine.

The findings of this investigation have some limitations. Firstly, about 40% of the initial sample from 2020 participated in all three rounds of measurement of SARS-CoV-2 antibodies. However, the rate of loss to follow-up was within the range expected for this type of study [21,26,27], and, assuming that the loss is random, this issue would not affect the representativeness of the sample. In fact, the sample size is adequate for statistical power and the biodemographic characteristics of the original sample were maintained among those who continued across all three rounds [5,11]. Secondly, our study does not differentiate between SARS-CoV-2 antibodies generated as a result of infection and vaccination. It is highly recommended that future seroprevalence studies include measurements of both anti-S and anti-N antibodies in order to separately assess whether the type of protection is natural or acquired by vaccination [19], especially in countries with high vaccination coverage, such as Chile. Thirdly, we did not measure neutralizing antibodies; this work is currently being carried out, and the results will be reported later. Finally, it was not possible to carry out a risk analysis based on sociodemographic or clinical variables, since the high prevalence of antibodies (i.e., only five seronegative individuals) made it impossible to perform subgroup comparisons.

## 5. Conclusions

The world-leading vaccination coverage in Chile enabled the maintenance of high rates of seroprevalence of antibodies against the SARS-CoV-2 virus in the population, regardless of demographic variables and social determinants of health. The seroprevalence was very low in the first round. The progression of the pandemic was evidenced by the increase in seropositivity from 46.2% in the second round to 85.7% in the third round among the few unvaccinated participants. Moreover, this study also demonstrates the importance of vaccination boosters to maintain immunity over time, which also depends on the type of vaccine previously administered.

## Figures and Tables

**Table 1:** Description of the characteristics of the participants in the three rounds of the seroprevalence study in two Chilean cities, 2020–2022.

Variable	Categories	*n*	%
City	Coquimbo-La Serena	184	47.7
	Talca	202	52.3
Sex	Male	126	32.6
(*n* = 386)	Female	260	67.4
Age at the third round	Under 10	4	1.0
(*n* = 386)	10–19	36	9.3
	20–29	41	10.6
	30–39	39	10.1
	40–49	65	16.8
	50–59	74	19.2
	60–69	66	17.1
	70 and over	61	15.8
Nationality	Chilean	384	99.5
(*n* = 386)	Foreign	2	0.5
Native Chilean ethnicity	No	360	93.3
(*n* = 386)	Yes	26	6.7
Education (*n* = 386)	Primary education or no formal education	68	17.6
	Secondary education	206	53.4
	High-level technical	34	8.8
	Professional	78	20.2
Education for participants over 18 years old	Primary education or no formal education	52	14.8
(*n* = 351)	Secondary education	187	53.3
	High-level technical	34	9.7
	Professional	78	22.2
Healthcare prevision	Social security (FONASA)	313	85.8
(*n* = 365)	Private	52	14.3
Face-to-face work	No	155	44.8
(*n* = 346)	Yes	191	55.2
Health worker	No	179	93.7
(*n* = 191)	Yes	12	6.3
Upper respiratory episodes	No	209	54.2
(*n* = 386)	Yes	177	45.9
Number of upper respiratory episodes	1 episode	84	47.5
(*n* = 177)	2 or more episodes	93	52.5
COVID-19 diagnosis	No	298	77.2
(*n* = 386)	Yes	88	22.8
Number of COVID-19 diagnoses	Once	86	97.3
(*n* = 88)	Twice	2	2.3
Symptomatology	None	211	54.7
(*n* = 386)	Yes	175	45.3
Nutritional status (body	Underweight	18	5.0
mass index)	Normal	95	26.5
(*n* = 359)	Overweight	135	37.6
	Obese	111	30.9
Comorbidities	No	153	39.6
(*n* = 386)	Yes	233	60.4
Tobacco smoker	No	283	73.3
(*n* = 386)	Yes	103	26.7
Vaccinated	No	6	1.6
(*n* = 386)	Yes	380	98.5
Vaccine doses	No	6	1.6
(*n* = 386)	1 dose	1	0.3
	2 doses or full baseline	27	7.0
	Baseline + 1 booster	213	55.2
	Baseline + 2 booster	139	36
Vaccine scheme	S	1	0.2
(*n* = 380)	P-P	7	1.8
	S-S	19	5.0
	Another baseline	1	0.3
	P-P-P	93	24.5
	S-S-P	82	21.6
	S-S-A	20	5.3
	Another baseline and 1 booster	18	4.8
	P-P-P-P	15	4.0
	P-P-P-M	10	2.6
	S-S-P-P	35	9.2
	S-S-A-P	58	15.0
	Another baseline and 2 boosters	21	5.5

S = Sinovac/CoronaVac; P = Pfizer; A = AstraZeneca; M = Moderna.

**Table 2:** Seroprevalence rates according to variables of interest in the participants in the three rounds.

Variable	Categories (*n* of Participants)	Round 1		Round 2		Round 3	
		September–November 2020		October–November 2021		April–May 2022	
		*n*	%	*n*	%	*n*	%
Global seroprevalence	(*n* = 386)	14	3.6	374	96.9	381	98.7
Global seroprevalence adjusted for test characteristics *	(*n* = 386)	14	3.7	374	-	381	98.7
Urban centre	Coquimbo—La Serena (*n* = 184)	9	4.9	174	94.6	183	99.5
	Talca (*n* = 202)	5	2.5	200	99.0	198	98.0
One COVID-19 infection	(*n* = 386)	8	47.1	34	100	87	98.9
Age	Under 10-years-old (*n =* 4)	0	0	2	50	4	100
	10–19 y. (*n =* 36)	0	0	33	91.7	36	100
	20–29 y. (*n =* 41)	1	2.5	41	100	40	97.6
	30–39 y. (*n =* 39)	1	2.6	39	100	38	97.4
	40–49 y. (*n =* 65)	5	7.7	63	96.9	65	100
	50–59 y. (*n =* 74)	3	4.1	73	98.7	74	100
	60–69 y. (*n* = 66)	4	6.1	64	97	66	100
	70 and over (*n* = 61)	0	0	59	96.7	58	95.1
Sex	Male (*n* = 126)	5	4.0	123	97.6	125	99.2
	Female (260)	9	3.5	251	96.5	256	98.5
Education among participants aged 18 years or older	Primary or no formal education (*n =* 52)	0	0	51	98.1	50	96.2
	High school (*n =* 187)	7	3.7	182	97.3	186	99.5
	Technical education (*n =* 34)	3	8.8	34	100	33	97.1
	Professional (*n =* 78)	4	5.1	77	98.7	77	98.7
Ethnic minority	Yes (*n =* 26)	2	7.7	26	100	26	100
Comorbidities	Yes (*n =* 233)	9	3.9	228	97.9	230	98.7
Vaccination scheme	No vaccine	-	-	6	46.2	5	83.3
	1 dose	-	-	7	77.8	1	100
	Full baseline	-	-	180	98.9	24	88.9
	Baseline + 1 booster	-	-	181	99.5	212	99.5
	Baseline + 2 booster	-	-	-	-	139	100

* First round: sensitivity = 99% and specificity = 100%; second round: sensitivity = 96.7 and specificity = 97.5%; third round: sensitivity = 100% and specificity = 99%.

**Table 3:** Doses of vaccines administered stratified by the time elapsed since last SARS-CoV-2 vaccine.

		Round 2 (October–November 2021)			Round 3 (April–May 2022)		
Vaccination Scheme	Time between Last Vaccine and Sample Collection According to Round	*N* (%) Samples	Seropositive (*n*)	%	*N* (%) Samples	Seropositive (*n*)	%
Not vaccinated		13 (3.4%)	6	46.2	6 (1.6%)	5	83.3
1 dose		9 (2.3%)	7	77.8	1 (0.3%)	1	100
	Less than 15 days	4	2	50	-	-	-
	15 to 179 days	5	5	100	1	1	100
	180 days and more	-	-	-	-	-	-
Full baseline		182 (47.2%)	180	98.9	26 (6.7%)	23	88.5
	Less than 15 days	3	3	100			
	15 to 179 days	131	129	98.5	14	14	100
	180 days and more	48	48	100	12	9	75
Baseline + 1 booster		182 (47.2%)	181	99.5	213 (55.2%)	212	99.5
	Less than 15 days	37	37	100	4	4	100
	15 to 179 days	145	144	99.3	147	147	100
	180 days and more	-	-	-	62	61	98.4
Baseline + 2 boosters		-	-	-	139 (36.0%)	139	100
	Less than 15 days	-	-	-	42	42	100
	15 to 179 days	-	-	-	96	96	100
	180 days and more	-	-	-	1	1	100

**Table 4:** SARS-CoV-2 antibody seroprevalence rates in each round of follow-up and average number of days between administration of the last vaccine and sample collection in each round.

Scheme (n)	N–P–P (%)	Average Days (Delta Round 2–Delta Round 3)	P–P–P (%)	Average Days (Delta Round 2–Delta Round 3)	N–P–N (%)	Average Days (Delta Round 2–Delta Round 3)	N–N–P (%)	Average Days (Delta Round 2–Delta Round 3)	N–N–N (%)
No vaccine (6)	3 (50)	-	-	-	-	-	2 (33.3)	-	1 (16.6)
Corona Vac (1)	1 (100)	(NV-179)	-	-	-	-	-	-	-
Pfizer-Pfizer (7)	6 (85.7)	(42.8–215.3)	-	-	-	-	1 (14.3)	(NV-44)	-
Corona Vac–Corona Vac (19)	12 (63.2)	(67.5–209.9)	-	-	2 (10.5)	(52.5–224)	5 (26.3)	(4.5–122)	-
AstraZeneca–AstraZeneca (1)	-	-	-	-	1 (100)	(130–303)	-	-	-
Pfizer–Pfizer–Pfizer (93)	89 (95.7)	(120.5–143.7)	3 (3.2)	(125.7–131)	1 (1.1)	(19–190)	-	-	-
Corona Vac–Corona Vac–Pfizer (82)	78 (95.1)	(104.2–156.6)	2 (2.4)	(57–166)	-	-	2 (2.4)	(75–96)	-
Corona Vac–Corona Vac–AstraZeneca (20)	18 (90)	(56.3–215.3)	2 (10)	(30–228)	-	-	-	-	-
Another baseline + 1 booster (18)	18 (100)	(108.8–131.8)	-	-	-	-	-	-	-
Pfizer–Pfizer–Pfizer–Pfizer (15)	15 (100)	(117.1–19.5)	-	-	-	-	-	-	-
Pfizer–Pfizer–Pfizer–Moderna (10)	8 (80)	(37.8–7.6)	2 (20)	(11.5–0)	-	-	-	-	-
Corona Vac–Corona Vac–AstraZeneca–Pfizer (58)	57 (98.3)	(67.3–57.1)	1 (1.7)	(70–13)	-	-	-	-	-
Corona Vac–Corona Vac–Pfizer–Pfizer (35)	32 (91.4)	(55.7–54.6)	3 (8.6)	(99.3–25)	-	-		-	-
Another baseline + 2 booster (21)	19 (90.5)	(51.6–39.6)	1 (4.8)	(23–15)	-	-	1 (4.8)	(73–55)	-

N–P–P = negative–positive–positive (most probably not vaccinated and not infected–recently vaccinated [or infected]–vaccinated or infected and maintained natural immune response); P–P–P = positive–positive–positive (most probably infected and maintained natural immune response or vaccinated–vaccinated or maintained natural immune response); N–P–N = negative–positive–negative (most probably not vaccinated and not infected–vaccinated or infected–loss of antibodies over time); N–N–P = negative–negative–positive (most probably not vaccinated and not infected in first and second rounds–recently vaccinated or infected at third round); N–N–N = negative–negative–negative (most probably never vaccinated and never infected); NV = no vaccine. Delta = (sample collection date-last administered vaccine).

## Data Availability

Not applicable.
